# Supplementary material for: Discovery of an ene-reductase for initiating flavone and flavonol catabolism in gut bacteria
Source: Nat Commun. 2021 Feb 4;12:790. doi: 10.1038/s41467-021-20974-2 (PMC7862272; doi:10.1038/s41467-021-20974-2)
Supplement: Supplementary file 1 — Supplementary information [file 41467_2021_20974_MOESM1_ESM.pdf]

Discovery of an ene-reductase for initiating flavone and flavonol catabolism in gut bacteria

Gaohua Yang[1, 2], Sen Hong[2, 3], Pengjie Yang[1, 2], Yuwei Sun[1], Yong Wang[1], Peng Zhang[3], Weihong Jiang[1]*, Yang Gu[1]*

[1]CAS-Key Laboratory of Synthetic Biology, CAS Center for Excellence in Molecular Plant Sciences, Shanghai Institute of Plant Physiology and Ecology, Chinese Academy of Sciences, Shanghai, 200032, China

[2]University of Chinese Academy of Sciences, Beijing, China

[3]National Key Laboratory of Plant Molecular Genetics, CAS Center for Excellence in Molecular Plant Sciences, Shanghai Institute of Plant Physiology and Ecology, Chinese Academy of Sciences, Shanghai, 200032, China

SUPPLEMENTARY INFORMATION

Supplementary Tables

Supplementary Table 1: The predicted 72 FLRs that are from 54 microbial strains

Gene tag	microbial species	habitat
cst_CLOST_0201	*Acetoanaerobium sticklandii*	Digestive tract
aalt_CC77DRAFT_1046349	*Alternaria alternata*	Plant
byl_A4V09_02065	*Blautia* sp. YL58	Digestive tract
bhp_BHAMNSH16_10850	*Brachyspira hampsonii*	Digestive tract
bhd_BHYOB78_03740	*Brachyspira hyodysenteriae* ATCC 27164	Digestive tract
bhy_BHWA1_02501	*Brachyspira hyodysenteriae* WA1	Digestive tract
bip_Bint_0725	*Brachyspira intermedia*	Digestive tract
brm_Bmur_2158	*Brachyspira murdochii*	Digestive tract
bpo_BP951000_0602	*Brachyspira pilosicoli* 95/1000	Digestive tract
bpj_B2904_orf763	*Brachyspira pilosicoli* B2904	Digestive tract
bpip_BPP43_12180	*Brachyspira pilosicoli* P43/6/78	Digestive tract
bpw_WESB_1903	*Brachyspira pilosicoli* WesB	Digestive tract
cale_FDN13_12325	*Caloramator* sp. E03	Aquatic
chy_CHY_1344	*Carboxydothermus hydrogenoformans*	Digestive tract
cdf_CD630_36370	*Clostridioides difficile* 630	Digestive tract
pdc_CDIF630_03961	*Clostridioides difficile* 630	Digestive tract
pdf_CD630DERM_36370	*Clostridioides difficile* 630Derm	Digestive tract
cdc_CD196_3450	*Clostridioides difficile* CD196	Digestive tract
cdl_CDR20291_3496	*Clostridioides difficile* R20291	Digestive tract
cac_CA_C2528	*Clostridium acetobutylicum* ATCC 824	Plants
cae_SMB_G2563	*Clostridium acetobutylicum* DSM 1731	Plants
cay_CEA_G2541	*Clostridium acetobutylicum* EA 2018	Plants
cah_CAETHG_1029	*Clostridium autoethanogenum*	Digestive tract
cbz_Cbs_3109	*Clostridium beijerinckii* ATCC 35702	Terrestrial
cbz_Cbs_3046	*Clostridium beijerinckii* ATCC 35702	Terrestrial
cbei_LF65_06425	*Clostridium beijerinckii* NCIMB 14988	Terrestrial
cbe_Cbei_3109	*Clostridium beijerinckii* NCIMB 8052	Digestive tract
cbe_Cbei_3046	*Clostridium beijerinckii* NCIMB 8052	Terrestrial
cbk_CLL_A0692	*Clostridium botulinum* B Eklund 17B	Digestive tract
cbut_ATN24_18485	*Clostridium butyricum*	Digestive tract
cbut_ATN24_18390	*Clostridium butyricum*	Digestive tract
cck_Ccar_15645	*Clostridium carboxidivorans*	Terrestrial
ccb_Clocel_4243	*Clostridium cellulovorans*	Plant
cdrk_B9W14_19690	*Clostridium drakei*	Terrestrial
ceu_A7L45_03445	*Clostridium estertheticum*	Terrestrial
ceu_A7L45_02165	*Clostridium estertheticum*	Terrestrial

clj_CLJU_c30220	*Clostridium ljungdahlii*	Digestive tract
csb_CLSA_c24800	*Clostridium saccharobutylicum*	Plant
csb_CLSA_c25700	*Clostridium saccharobutylicum*	Plant
csr_Cspa_c29810	*Clostridium saccharoperbutylacetonicum*	Terrestrial
csr_Cspa_c29950	*Clostridium saccharoperbutylacetonicum*	Terrestrial
csr_Cspa_c43180	*Clostridium saccharoperbutylacetonicum*	Terrestrial
csr_Cspa_c42860	*Clostridium saccharoperbutylacetonicum*	Terrestrial
csr_Cspa_c17360	*Clostridium saccharoperbutylacetonicum*	Terrestrial
csr_Cspa_c47530	*Clostridium saccharoperbutylacetonicum*	Terrestrial
csq_CSCA_3810	*Clostridium scatologenes*	Terrestrial
csq_CSCA_5025	*Clostridium scatologenes*	Terrestrial
csq_CSCA_1099	*Clostridium scatologenes*	Terrestrial
cls_CXIVA_16290	*Clostridium* sp. SY8519	Digestive tract
cls_CXIVA_10390	*Clostridium* sp. SY8519	Digestive tract
cls_CXIVA_19200	*Clostridium* sp. SY8519	Digestive tract
cls_CXIVA_14850	*Clostridium* sp. SY8519	Digestive tract
cck_Ccar_24190	*Clostridium carboxidivorans*	Digestive tract
cdrk_B9W14_00510	*Clostridium drakei*	Terrestrial
dgi_Desgi_3230	*Desulfallas gibsoniae*	Terrestrial
dku_Desku_1189	*Desulfofundulus kuznetsovii*	Digestive tract
ela_UCREL1_8140	*Eutypa lata*	Plant
fpla_A4U99_05915	*Flavonifractor plautii*	Digestive tract
fpla_A4U99_14100	*Flavonifractor plautii*	Digestive tract
fmo_C4N19_05200	*Fusobacterium mortiferum* ATCC 9817	Digestive tract
ful_C4N20_10740	*Fusobacterium ulcerans*	Digestive tract
fva_FV113G1_25840	*Fusobacterium varium*	Digestive tract
mdv_C5Q96_02325	*Mogibacterium diversum*	Digestive tract
ppeo_ABE82_12665	*Paenibacillus peoriae*	Terrestrial
ppol_X809_28335	*Paenibacillus polymyxa* CR1	Terrestrial
pfy_PFICI_12236	*Pestalotiopsis fici*	Plant
ral_Rumal_0329	*Ruminococcus albus*	Digestive tract
str_Sterm_3308	*Sebaldella termitidis*	Digestive tract
sge_DWG14_08345	*Streptomyces griseorubiginosus*	Terrestrial
scb_SCAB_23591	*Streptomyces scabiei*	Digestive tract
tep_TepiRe1_2235	*Tepidanaerobacter acetatoxydans* Re1	Digestive tract
tae_TepiRe1_2403	*Tepidanaerobacter acetatoxydans* Re1	Digestive tract

Supplementary Table 2: The BLASTP analyses of the *F. plauti* FLR and some representative ene-reductases reported previously

Gene	EC number	Enzyme names	Organism	Coverage	e-value	Identity	Sources
yqjM	1.6.99.1	OYE	*Bacillus subtilis*	6%	4.3	39.13%	Brenda
xenA	1.6.99.1	OYE	*Pseudomonas putida*	12%	1.3	54.55%	Brenda
ERED	1.3.1.31	EnoR	*Achromobacter* sp. JA81	10%	0.27	57.14%	Brenda
fldZ	1.3.1.31	EnoR	*Clostridium tyrobutyricum*	29%	0.30	32.35%	Brenda
AER	1.3.1.74	MDR	*Arabidopsis thaliana*	25%	2.0	23.30%	Brenda
PTGR	1.3.1.48	MDR	*Homo sapiens*	51%	0.89	46.15%	Brenda
SDR2	1.1.1.208	SDR	*Capsicum annuum*	24%	0.48	26.92%	Brenda
SDR2	1.1.1.208	SDR	*Mentha piperita*	21%	1.1	28.57%	Brenda
TtENR	-	QnoR	*Thermus thermophilus*	13%	1.6	27.59%	1
PhENR	-	QnoR	*Pyrococcus horikoshii*	12%	2.1	25.00%	1

OYE: old yellow enzyme; EnoR: enoate reductase; MDR: medium-chain dehydrogenases/reductases; SDR: short-chain dehydrogenase/reductase; QnoR: NADPH-dependent quinone reductases; Brenda: The Comprehensive Enzyme Information System, https:// brenda-enzymes.org/.

Supplementary Table 3: Data collection and model refinement statistics

	SeMet-FLR	SeMet-FLR-Chrysin	SeMet-FLR-Apigenin	SeMet-FLR-Luteolin
Data collection				
Wavelength (Å)	0.97850	0.97849	0.97849	0.97849
Space group	$P4_32_12$	$P4_32_12$	$P4_32_12$	$P4_32_12$
Cell dimensions				
a, b, c (Å)	67.870, 67.870, 194.270	66.365, 66.365, 194.057	63.969, 63.969, 192.262	65.465, 65.465, 193.544
α, β, γ (°)	90, 90, 90	90, 90, 90	90, 90, 90	90, 90, 90
Number of molecules in ASU	1	1	1	1
Resolution (Å)	30-2.2 (2.28-2.20)[a]	30-2.65 (2.74-2.65)	30-2.55 (2.64-2.55)	30-2.25 (2.33-2.25)
Unique reflections	21222	11627	12318	16462
Redundancy	22.0	21.8	23.1	11.6
I/sigma (I)	34.5(2.33)	30(3.14)	28.67(2.15)	24.4(2)
R_{merge}	0.110(0.522)	0.136(0.701)	0.149(0.870)	0.099(0.970)
Completeness (%)	99.6(96.7)	99.7(97.1)	99.9(99.3)	100(99.8)
$CC_{1/2}$	0.973	0.990	0.949	0.995
Refinement				
R_{work}/R_{free}[b]	0.1811/0.2107	0.1903/0.2478	0.1942/0.2579	0.1947/0.2549
Number of atoms	2419	2309	2315	2382
Ligands	34	53	54	55
Protein residues	290	290	290	290
R.m.s.d				
Bond lengths (Å)	0.009	0.009	0.009	0.009
Bond angles (°)	0.899	1.013	0.976	0.959
Ramachandran				
Favored (%)	97.54	95.07	96.13	96.83
Allowed (%)	2.46	4.93	3.87	3.17
Average B factor	33.39	41.41	38.60	37.49
Protein	32.87	41.59	38.79	37.48
Ligand	28.91	35.50	33.79	34.42
Water	41.46	39.31	34.66	39.39

[a] Numbers in parentheses are for highest-resolution shell

[b] $R = \Sigma_{hkl}||F_{obs}|-|F_{cal}||/\Sigma_{hkl}|F_{obs}|$, where F_{obs}andF_{calc} are observed and calculated structure-factor amplitudes, respectively. R_{free} was calculated as for$R$but using a subset (10%) of reflections that were not used for refinement.

Supplementary Table 4: Primers used in this work

Primer name	Sequence (5′-3′)
A4U99_05915-s	CTAGCTAGCatgaaaattttgggtatttccgg
A4U99_05915-a	CGCGGATCCtcagcccttctcgggcacggt
CoFLR-tru5-a	CGGGATCCtcagcggtacgcggcctggcgggcc
CoFLR-G106A-s	ccacacgattaccgaccgctttgcccccccgcatggaccgcggcaac
CoFLR-G106A-a	gttgccgcggtccatgcgggggggcaaagcggtcggtaatcgtgtgg
CoFLR-P107A-s	cacacgattaccgaccgctttggcgcccgcatggaccgcggcaacaac
CoFLR-P107A-a	gttgttgccgcggtccatgcgggcgccaaagcggtcggtaatcgtgtg
CoFLR-G146A-s	catctccttcatgtccgtgggcgcctccgactgggtgacccgcaccc
CoFLR-G146A-a	gggtgcgggtcacccagtcggaggcgcccacggacatgaaggagatg
CoFLR-S147A-s	ctccttcatgtccgtgggcggcgccgactgggtgacccgcacccag
CoFLR-S147A-a	ctgggtgcgggtcacccagtcggcgccgcccacggacatgaaggag
CoFLR-W149A-s	ccttcatgtccgtgggcggctccgacgcggtgacccgcacccagtgcgacg
CoFLR-W149A-a	cgtcgcactgggtgcgggtcaccgcgtcggagccgcccacggacatgaagg
CoFLR-L178A-s	caacgaggtgttcccttgggcggcgtccatcctggtggaggacgag
CoFLR-L178A-a	ctcgtcctccaccaggatggacgccgcccaagggaacacctcgttg
CoFLR-H271A-s	caccctgtccggcaagttcatcgccggcaacgacatcaaggagaatac
CoFLR-H271A-a	gtattctccttgatgtcgttgccggcgatgaacttgccggacagggtg
CoFLR-I275A-s	caagttcatccacggcaacgacgccaaggagaataccggcaagaag
CoFLR-I275A-a	cttcttgccggtattctccttggcgtcgttgccgtggatgaacttg
30220-gRNA-s	taaggaggagttttcgtcgaccatagattgccatgaacgtggttttagagctagaaata
30220-gRNA-a	ataaaaataagaagcctgcaaatgcagg
30220-up-s	tttgcaggcttcttatttttattggcattcaataggaagtgatctg
30220-up-a	ccttacttttcctcctaaataaatttc
30220-down-s	aatttatttaggaggaaaagtaaggttttatattgaatcaaaattcccaaggt
30220-down-a	agcttgcatgtctgcaggccccggggacacaaatagaaattttactgatttg
30220-pr-s	tcaggttggatactgtgtagccca
30220-pr-a	ggagaaaatttttgaagcaaaaagtgc
P1440-s	gattacgaattcgagctcggtaccggcattttcaaagaaataactagtc
P1440-a	gaaatacccaaaattttcatggatcccttatcctccttaatttttagataatg
CoFLR-s	atgaaaattttgggtatttccgg
CoFLR-a	cgtcgactctagaagatctcccgggtcagcccttctcgggcacggtggcg
ClFLR-s	gattacgaattcgagctcggtaccacttcatcaccaatctttgcacttttatg
ClFLR-a	cttgcatgtctgcaggcctcgagttatatagttttaaattgtatt
clj-qPCR-s	agatttaggaagagctgaatctgta
clj-qPCR-a	cagttgctgctccaacttttac
csci-qPCR-s	gaaaagagatgctcaaagatatcgc
csci-qPCR-a	ctttactttgctggacatcttagc
bvu-qPCR-s	agctactcttgaaatgttgggt
bvu-qPCR-a	acctacccctacgattgattgag
bbf-qPCR-s	gccttgaccttgagttcacc
bbf-qPCR-a	aaccttgccggaggtcag
csym-qPCR-s	gaagaactgggccttgatttg

csym-qPCR-a	ttcttaaaacgattgggttagcg
robe-qPCR-s	agcttggacgtgcaaaatcc
robe-qPCR-a	actgattttctggctcatctctttg
amu-qPCR-s	gctgcttgaaaaagtggc
amu-qPCR-a	ggcttcaatatcggaagatttgg
ere-qPCR-s	tcagttaaggttcagaaggagaaca
ere-qPCR-a	cacgacgaagcacgattg
cld-qPCR-s	aaaacttcaagaaagacttgctaaat
cld-qPCR-a	cagaagttaaatcagcaattttaggt

Supplementary Table 5: Plasmids used in this work

Plasmids	Relevant Characteristics	Sources or references
pET28a	Km^r; T7 promoter	Novagen
pET28a-A4U99_05915	Km^r; T7 promoter, expressing the A4U99_05915 gene	This study
pET28a-CoFLR-G106A	Kmr; T7 promoter, expressing the mutated *flr* gene (G106A)	This study
pET28a-CoFLR-P107A	Kmr; T7 promoter, expressing the mutated *flr* gene (P107A)	This study
pET28a-CoFLR-G146A	Kmr; T7 promoter, expressing the mutated *flr* gene (G146A)	This study
pET28a-CoFLR-S147A	Kmr; T7 promoter, expressing the mutated *flr* gene (S147A)	This study
pET28a-CoFLR-W149A	Kmr; T7 promoter, expressing the mutated *flr* gene (W149A)	This study
pET28a-CoFLR-L178A	Kmr; T7 promoter, expressing the mutated *flr* gene (L178A)	This study
pET28a-CoFLR-H271A	Kmr; T7 promoter, expressing the mutated *flr* gene (H271A)	This study
pET28a-CoFLR-I275A	Kmr; T7 promoter, expressing the mutated *flr* gene (I275A)	This study
pET28a-CoFLR-truncated-5	Km^r; T7 promoter, pET28a-CoFLR-truncated-5	This study
pMTL83151	*ColE1, catP, pCB102 ori*	
pMTLcas-clju_c 30220	Plasmid for clju_c 30220 deletion; ColE1, catP, pCB102 ori, tra, thl-Cas9, P_{araE}-sgRNA, homologous arms	This study
pMTL83151-ClFLR	pMTL83151-derived plasmid for expressing *C. ljungdahlii* flavone reductase	This study
pMTL83151-p1440-CoFLR	pMTL83151-derived plasmid for expressing *F. plautii* flavone reductase using the promoter P_{1440}	This study

Supplementary Figures

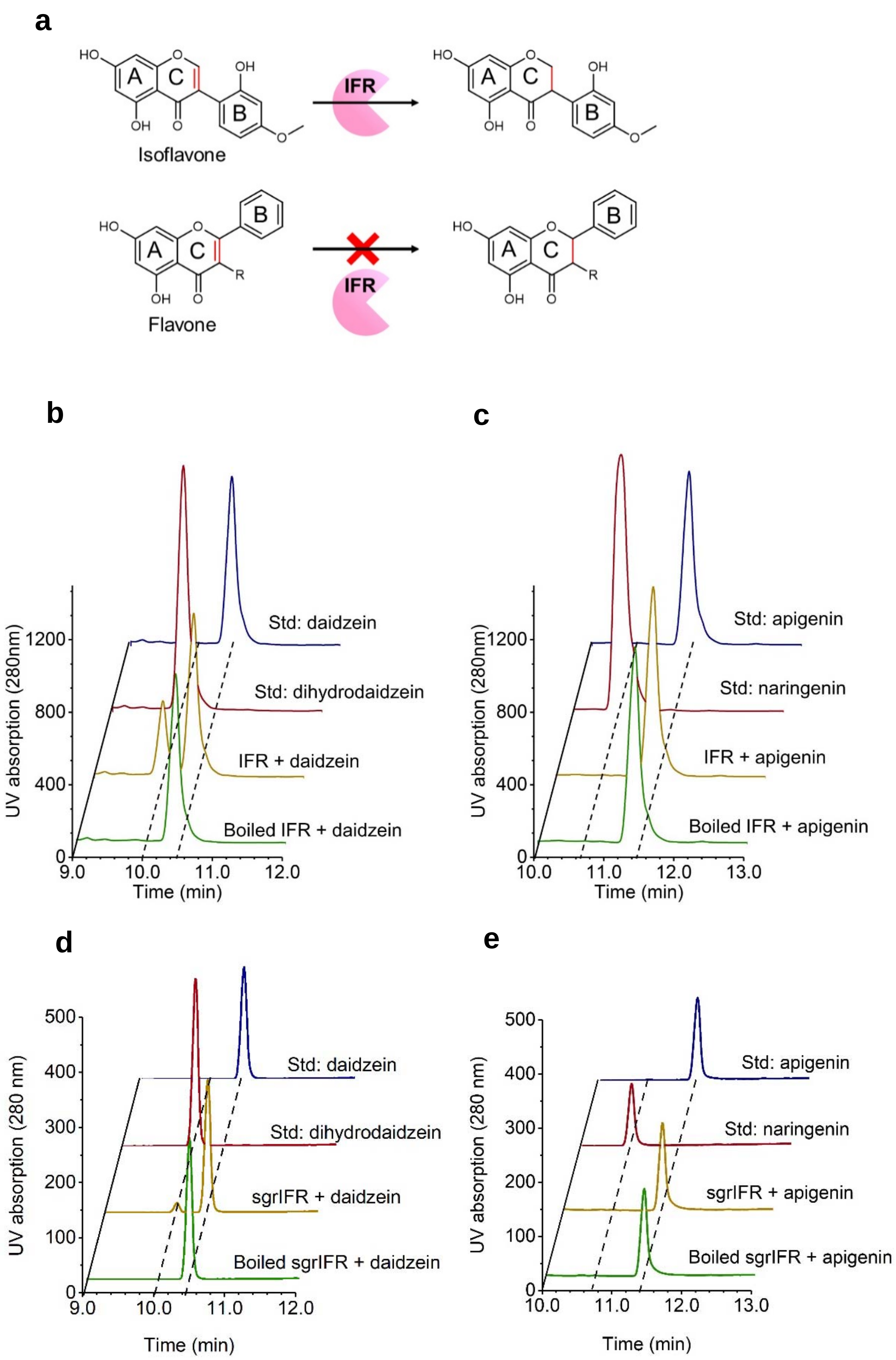

Supplementary Figure 1 *In vitro* activity analysis of the IFR enzyme (soybean isoflavone reductase, GmIFR). a IFR can catalyse the reduction of C2−C3 double bond of isoflavone, but not that of flavone. IFR, isoflavone reductase. **b** HPLC detection of daidzein (a major isoflavone) transformation to dihydrodaidzein by the GmIFR protein. **c** HPLC measurement determines that GmIFR has no catalytic activity towards apigenin. **d** HPLC detection of daidzein (a major isoflavone) transformation to dihydrodaidzein by the sgrIFR protein (gene ID: SGR_2256) from *Streptomyces griseus*. **e** HPLC measurement determines that sgrIFR has no catalytic activity towards apigenin. Std, standard samples.

a

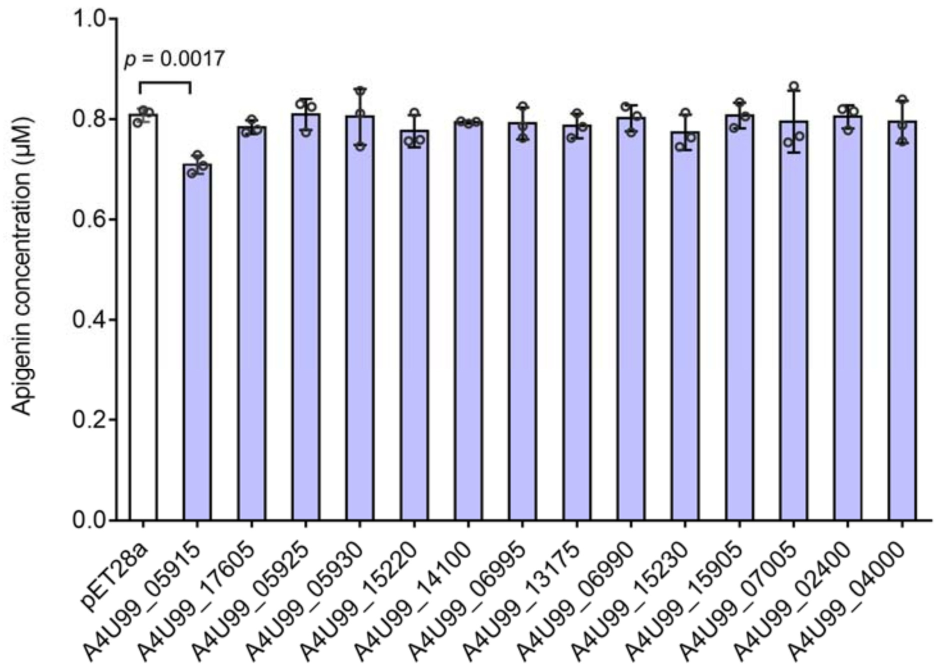

b

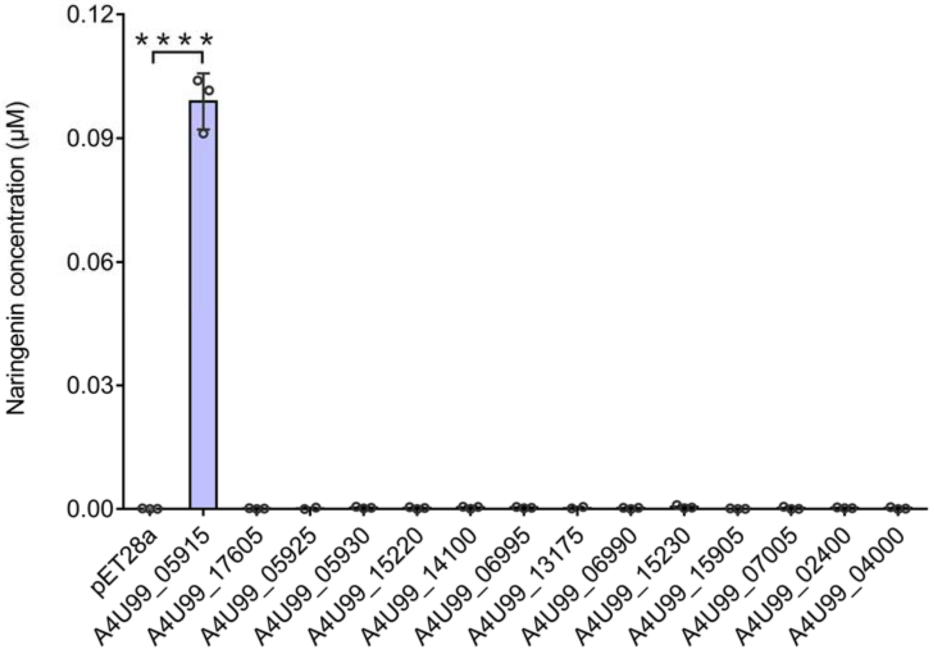

Supplementary Figure 2 Phenotypic screens reveal the potential flavone reductase. a apigenin consumption by the *E. coli* BL21 strains that express predicted enzymes. **b** naringenin production by the *E. coli* BL21 strains that express predicted

enzymes. The *E. coli* strains were anaerobically cultivated in the medium supplemented with 0.8 μM apigenin. The residual apigenin was measure by HPLC. Data are represented as the mean ± SD (n = 3). Error bars show SDs. Statistical analysis was performed by a two-tailed Student's *t*-test. ****, $P < 0.0001$.

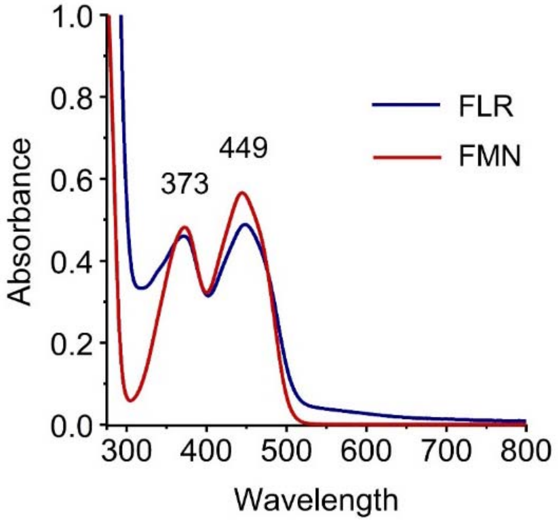

Supplementary Figure 3 Ultraviolet-visible spectra of the flavin mononucleotide (FMN) standard and purified FLR protein. The two characteristic absorption peaks at 373 and 449 nm for FLR supports the presence of FMN as the cofactor in FLR.

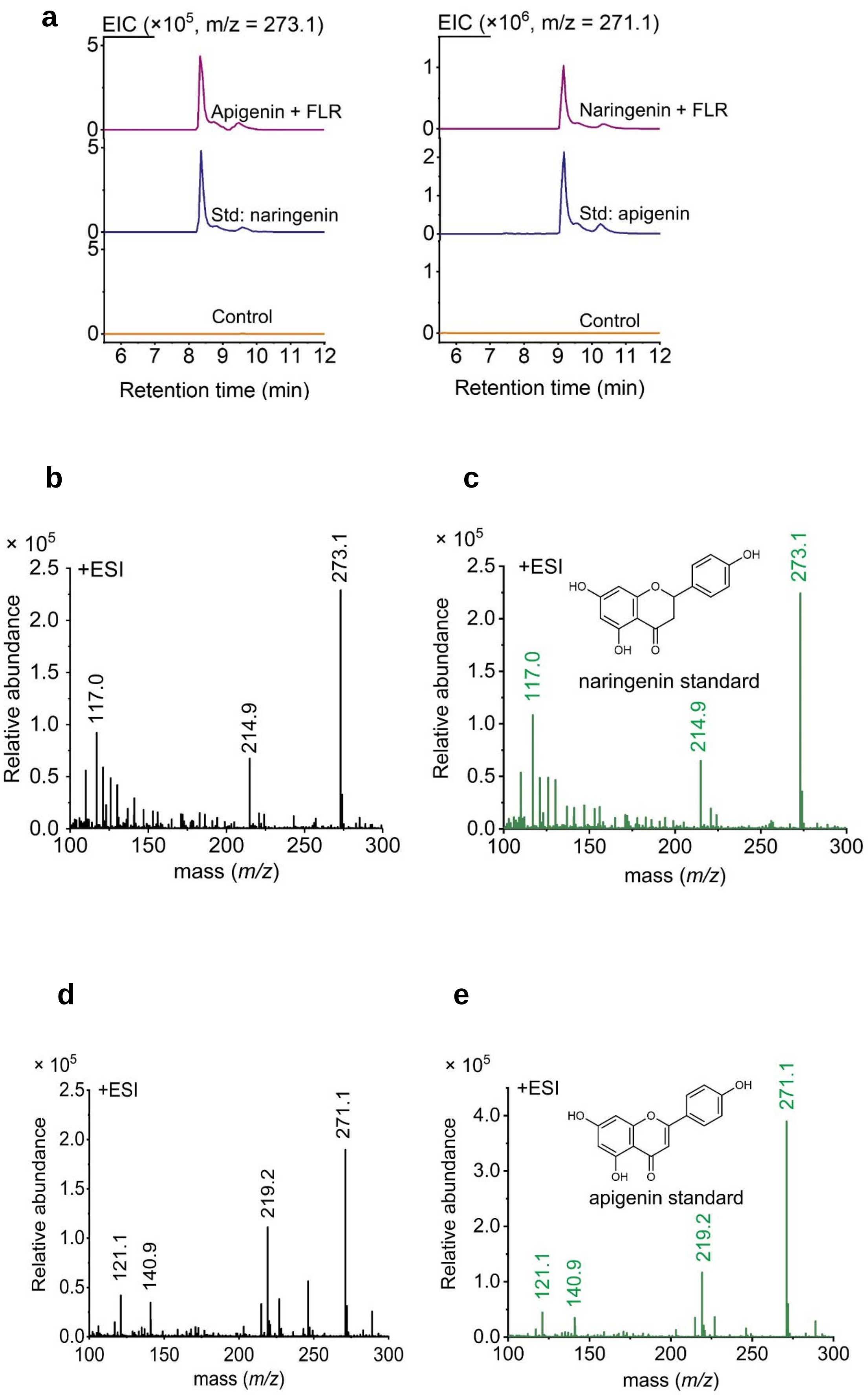
a
EIC (×10^5, m/z = 273.1)
Apigenin + FLR
Std: naringenin
Control
Retention time (min)
EIC (×10^6, m/z = 271.1)
Naringenin + FLR
Std: apigenin
Control
Retention time (min)
b
× 10^5
+ESI
273.1
117.0
214.9
Relative abundance
mass (m/z)
c
× 10^5
+ESI
273.1
117.0
214.9
naringenin standard
Relative abundance
mass (m/z)
d
× 10^5
+ESI
271.1
219.2
121.1
140.9
Relative abundance
mass (m/z)
e
× 10^5
+ESI
271.1
219.2
121.1
140.9
apigenin standard
Relative abundance
mass (m/z)

Supplementary Figure 4 Mass spectrometry identification of FLR-mediated transformation between naringenin and apigenin. a LC–MS detection of products (apigenin: *m/z* 271.1 $[M+H]^+$; naringenin: *m/z* 273.1 $[M+H]^+$) after incubation of apigenin or naringenin with the FLR enzyme. Experiments were repeated at least three times with similar results. **b, c** Tandem mass spectrometry analysis of the HPLC fractions corresponding to the main product (**b**) formed by incubation of FLR with apigenin *in vitro*, and comparison with that of the naringenin standard (**c**). **d, e** Tandem mass spectrometry analysis of the HPLC fractions corresponding to the main product (**d**) formed by incubation of FLR with naringenin *in vitro*, and comparison with that of the apigenin standard (**e**).

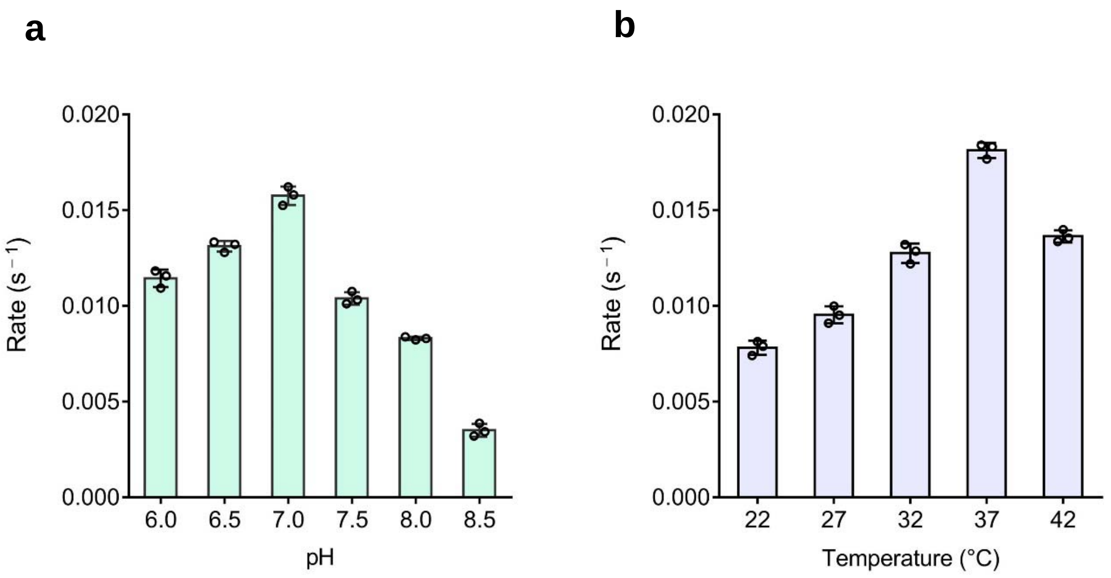

Supplementary Figure 5 Determination of the optimum reaction temperature and pH value for steady state kinetic analysis of FLR using apigenin as the substrate. a Determination of the optimum pH value. **b** Determination of the optimum reaction temperature. Data are represented as the mean ± SD (n = 3). Error bars show SDs.

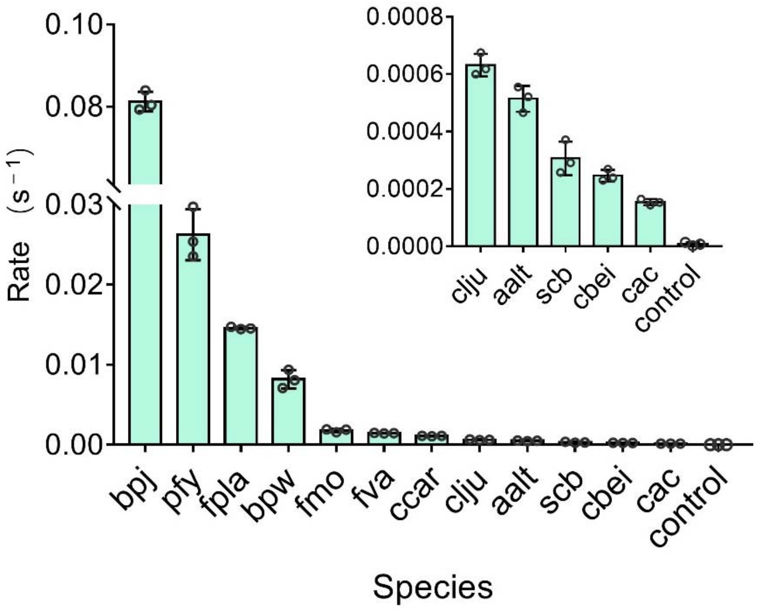

Abbr.	Organism	Gene	Abbr.	Organism	Gene
bpj	*Brachyspira pilosicoli* B2904	B2904_orf763	ccar	*Clostridium carboxidivorans*	Ccar_15645
pfy	*Pestalotiopsis fici*	PFICI_12236	clju	*Clostridium ljungdahlii*	CLJU_c30220
fpla	*Flavonifractor plautii*	A4U99_05915	aalt	*Alternaria alternata*	CC77DRAFT_1046349
bpw	*Brachyspira pilosicoli* WesB	WESB_1903	scb	*Streptomyces scabiei*	SCAB_23591
fmo	*Fusobacterium mortiferum*	C4N19_05200	cbei	*Clostridium beijerinckii* NCIMB 8052	Cbei_3109
fva	*Fusobacterium varium*	FV113G1_25840	cac	*Clostridium acetobutylicum* ATCC 824	CA_C2528

Supplementary Figure 6 *In vitro* activity analysis of the *Flavonifractor plautii* FLR and its 11 homologs from different microbial hosts using apigenin as the substrate. Control: an annotated isoflavone reductase (IFR) (sgrIFR, gene ID: SGR_2256) from *Streptomyces griseus*. The corresponding genes for these enzymes are listed in the table. Data are represented as the mean ± SD (n = 3). Error bars show SDs.

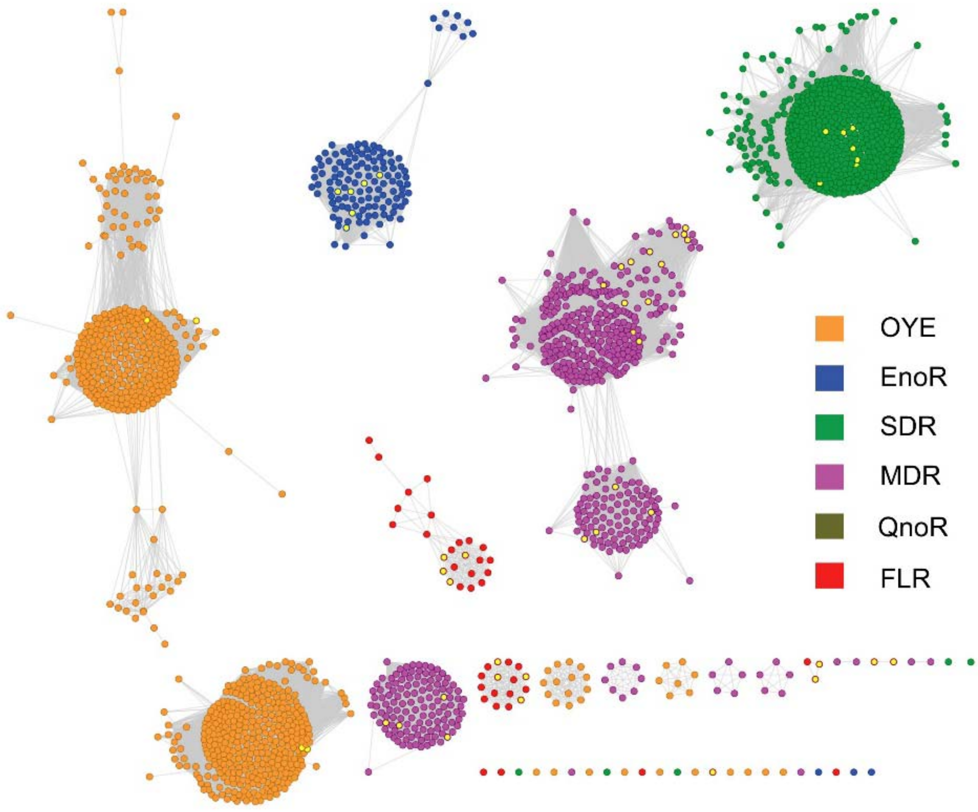

Supplementary Figure 7 The ene-reductase SSN with a higher edge score generated with an initial score of 10^{-20}. The “alignment score threshold” (that is a measure of the minimum sequence similarity threshold) for drawing the edges that connect the proteins (nodes) in the SSN was then refined such that nodes are connected by an edge if this value is ≥60. Each of the nodes within the SSN represents proteins with ≥90% amino acid identity and was colored according to the cluster type (OYE, EnoR, SDR, MDR, QnoR, and FLR). Each type of ene-reductase, as shown in a different colour, is separated into different clusters that may contain enzymes with similar biochemical activity. A total of 72 different FLR-like proteins which are shown in the phylogenic tree are denoted in red. The nodes representing the reported ene-reductase with enzyme activity data (from BRENDA Enzyme Database) and biochemically

identified FLR-like enzymes in this study were highlighted with lemon yellow. OYE, Old-Yellow-Enzyme; EnoR, oxygensensitive enoate reductases; SDR, short-chain dehydrogenase/reductase; MDR, medium-chain dehydrogenase/reductase; QnoR, quinone reductase-like ene-reductase.

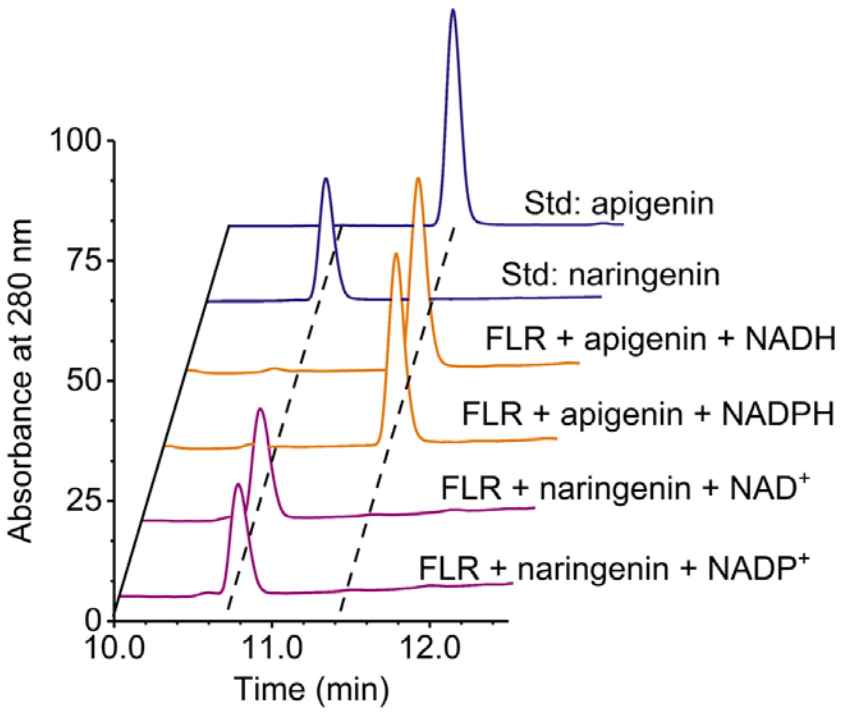

Supplementary Figure 8 *In vitro* activity analysis confirming that FLR is not an NAD(P)H-dependent reductase. Std, standard samples.

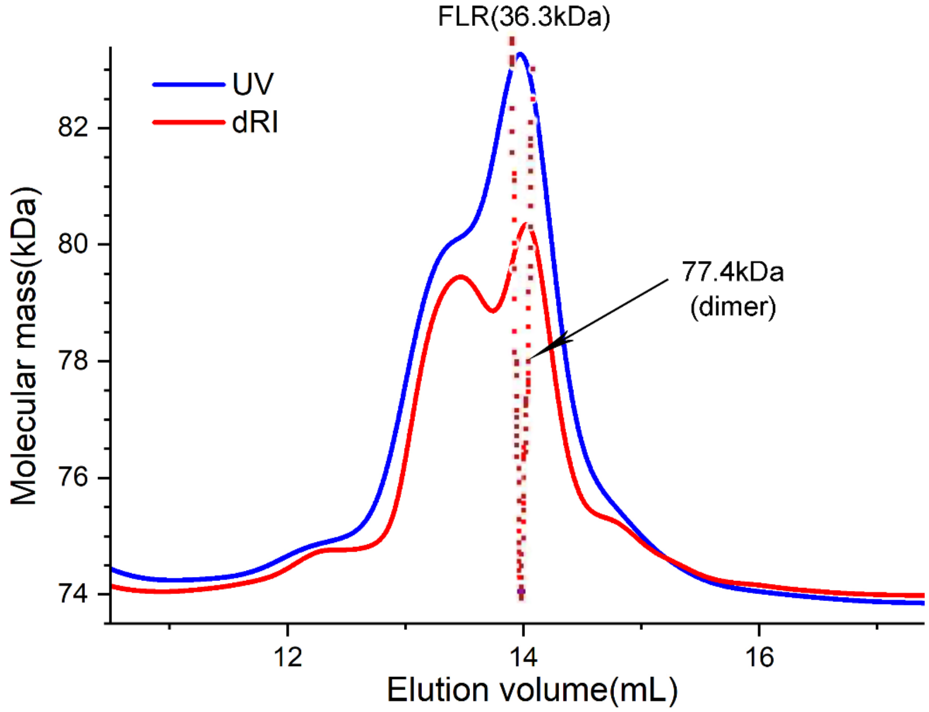

Supplementary Figure 9 The detection of FLR by Size Exclusion Chromatography-Multi Angle Laser Light Scattering (SEC-MALLS). Absorbance UV280 (UV) and differential refractive index (dRI) are showed in blue and red, respectively. The red dotted lines show the calculated molecular mass of protein sample purified from a Superdex-200 10/300 column. The theoretical sequence weight of protein is shown in parentheses.

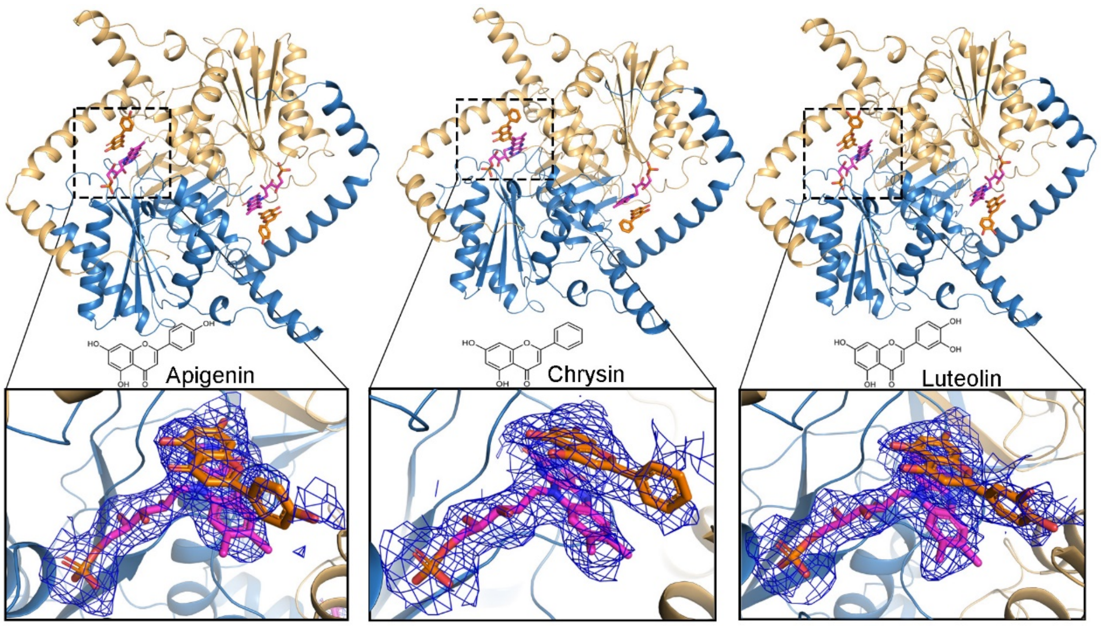

Supplementary Figure 10 The FLR's structural difference around the substrate-binding pocket in the presence of different substrates. The lower panels are zoom-in views of substrates-binding site. The pictures show *Fo-Fc* density map (blue), contoured at 1.0 σ level, from which the co-factors and substrates were omitted.

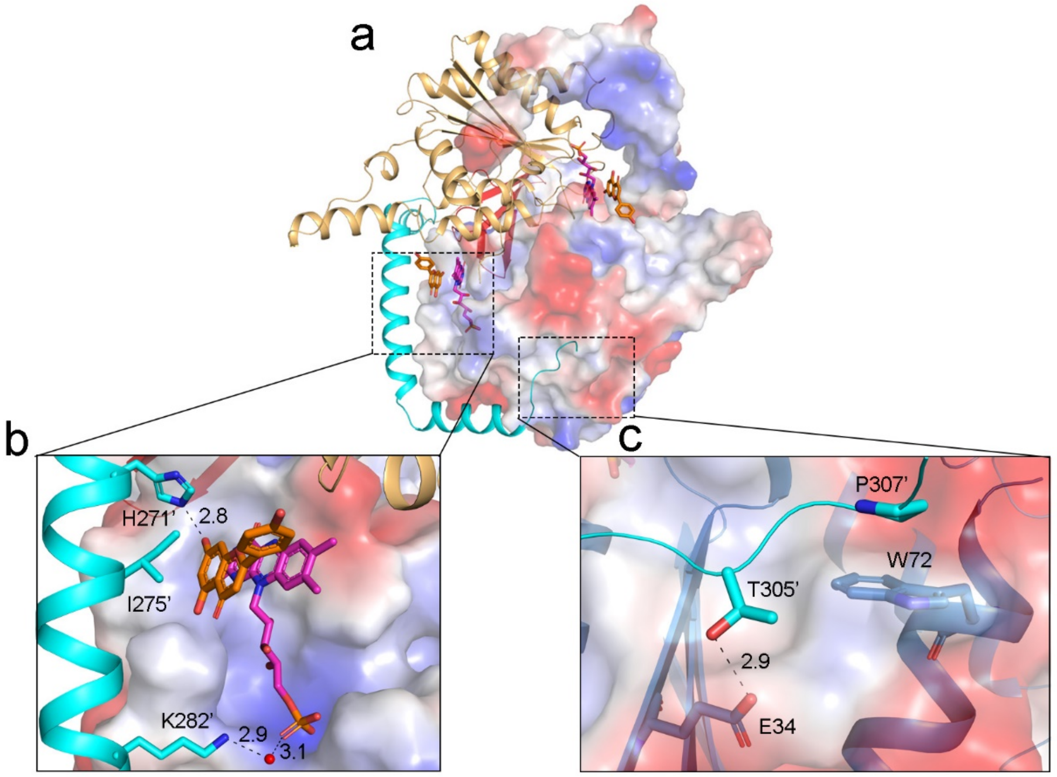

Supplementary Figure 11 Crystal structure of FLR dimmer and its interface. a Monomers A and B are shown with ribbon cartoon and electron static surface potential, respectively. The tail is colored cyan. **b, c** Zoom in view of amino acid residues interactions at interface. Water is shown as a sphere (red). Distances are shown by dashed lines.

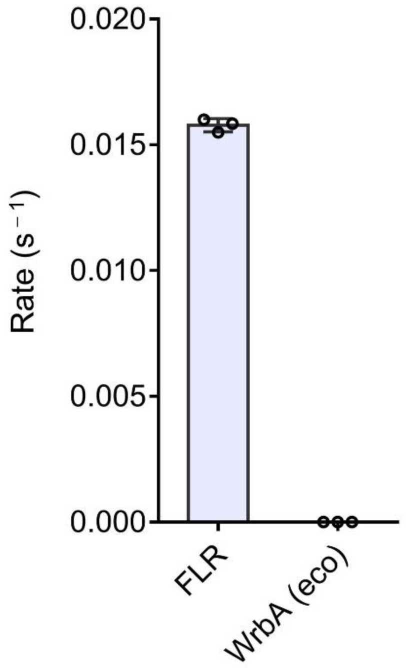

Supplementary Figure 12 Enzymatic assay confirms that the *E. coli* WrbA has no catalytic activity towards apigenin. WrbA (eco), the WrbA protein from *E. coli*. Data are represented as the mean ± SD (n = 3). Error bars show SDs.

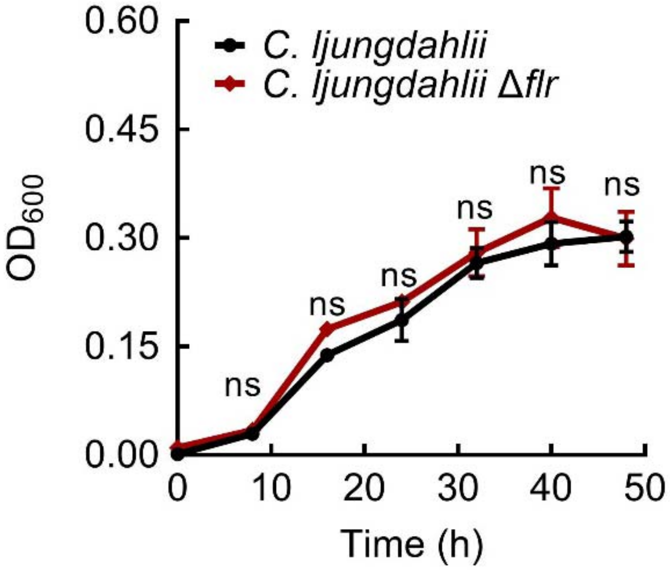

Supplementary Figure 13 Comparison of the growth rate of the wild-type *C. ljungdahlii* DSM 13528 (WT *C. ljungdahlii*) and the *C. ljungdahlii* Δ*flr* mutant (in the absence of flavones and flavonols). Data are presented as the means ± SD (*n* = 3). Error bars show SDs. Statistical analysis was performed by a two-tailed Student's *t*-test. ns, no significance.

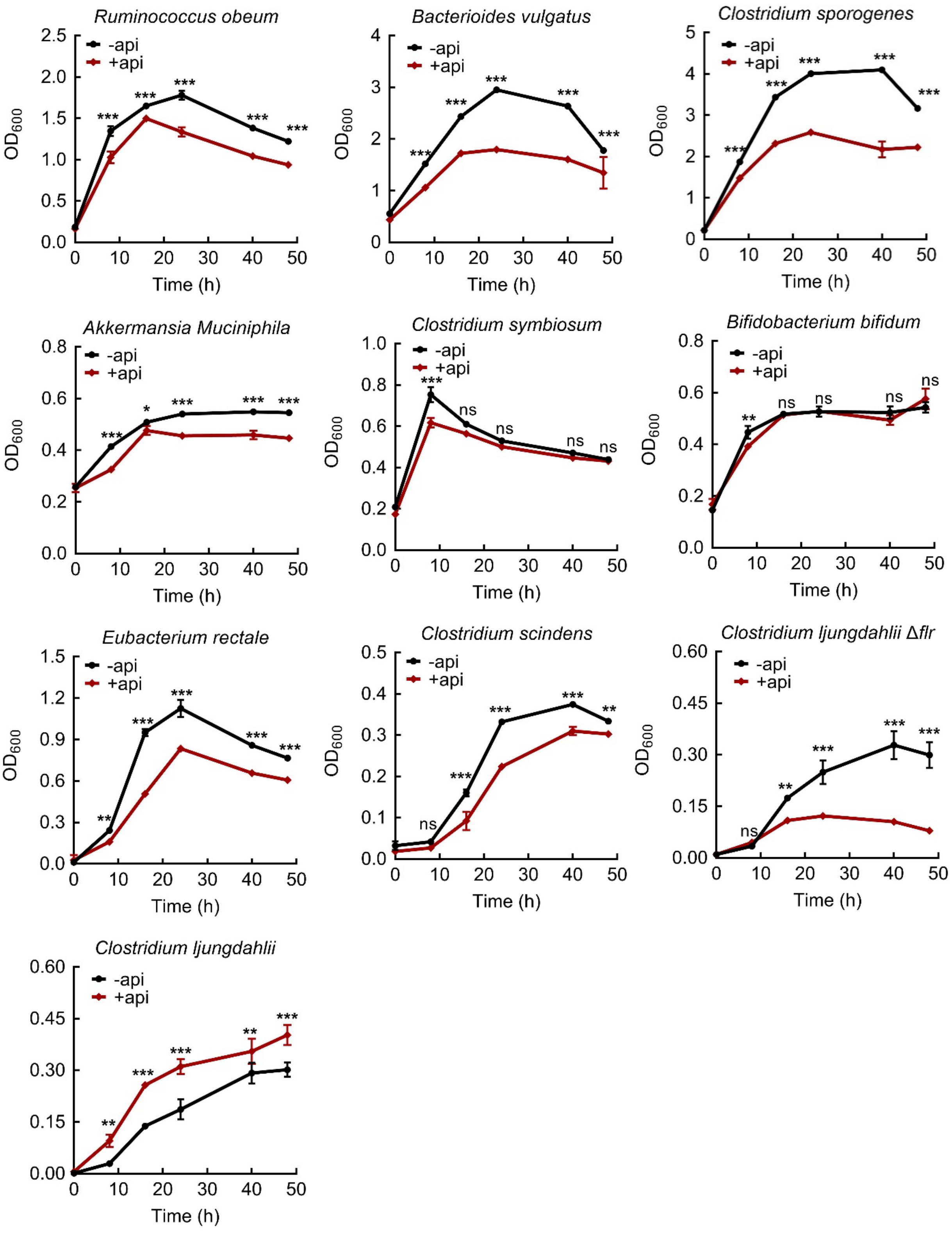

Supplementary Figure 14 The effects of apigenin on the growth of each individual isolates in the artificially constructed gut microbial community. Data are presented as the means ± SD ($n = 3$). Error bars show SDs. Statistical analysis was

performed by a two-tailed Student's *t*-test. *, $P < 0.05$; **, $P < 0.01$; ***, $P < 0.001$; ns, no significance.

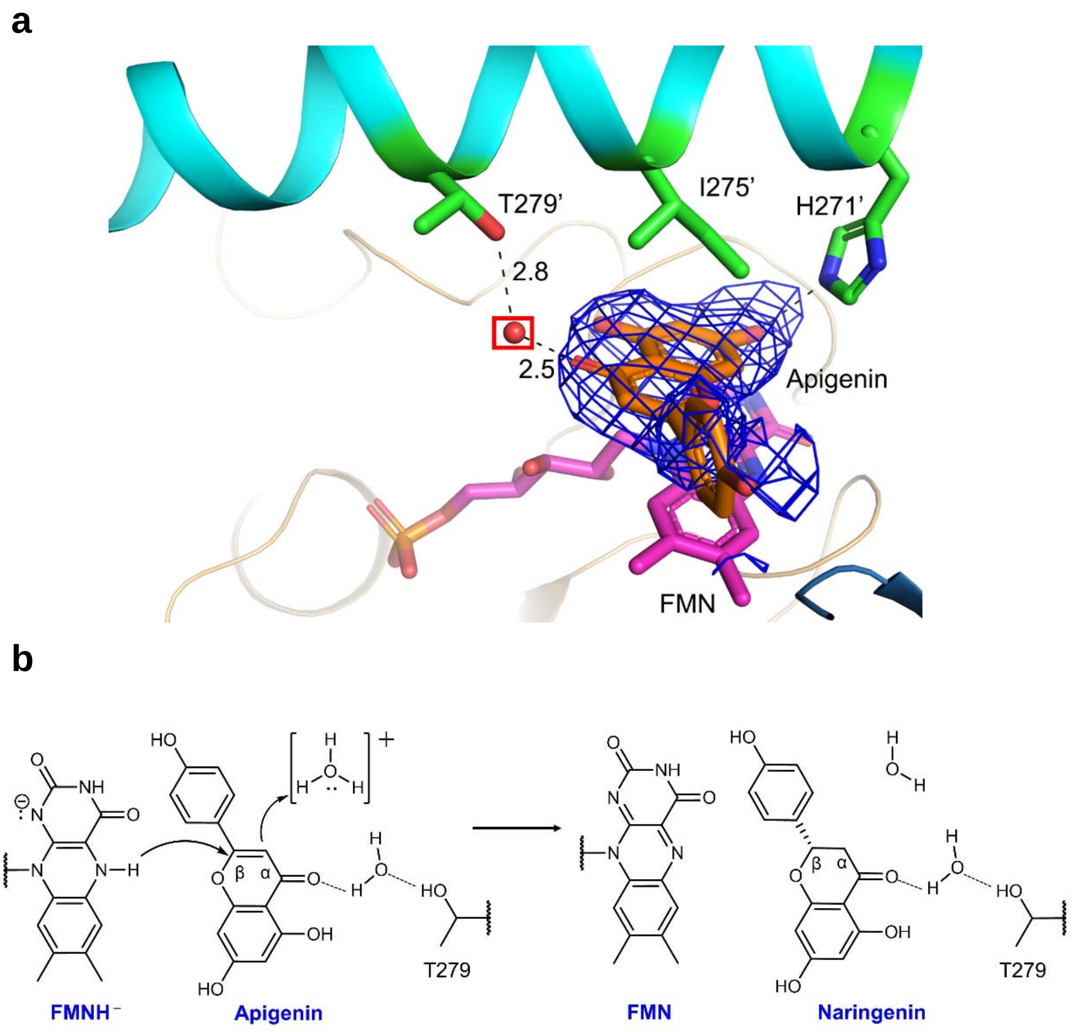

Supplementary Figure 15 Comparison of FLRs and known Old-Yellow-Enzymes regarding active site composition and catalytic mechanism. a Binding of apigenin to the FMN site of FLR. Amino acid residues surrounding the active site are shown with sticks. The picture shows *Fo-Fc* density map (blue), contoured at 1.0 σ level, from which the apigenin molecule was omitted. The water molecule (highlighted with a red box) that lies between the Thr279 residue of FLR and the C4-carbonyl group of apigenin may activate the substrate (apigenin). **b** Possible mechanism of the FLR-catalyzed apigenin reduction.

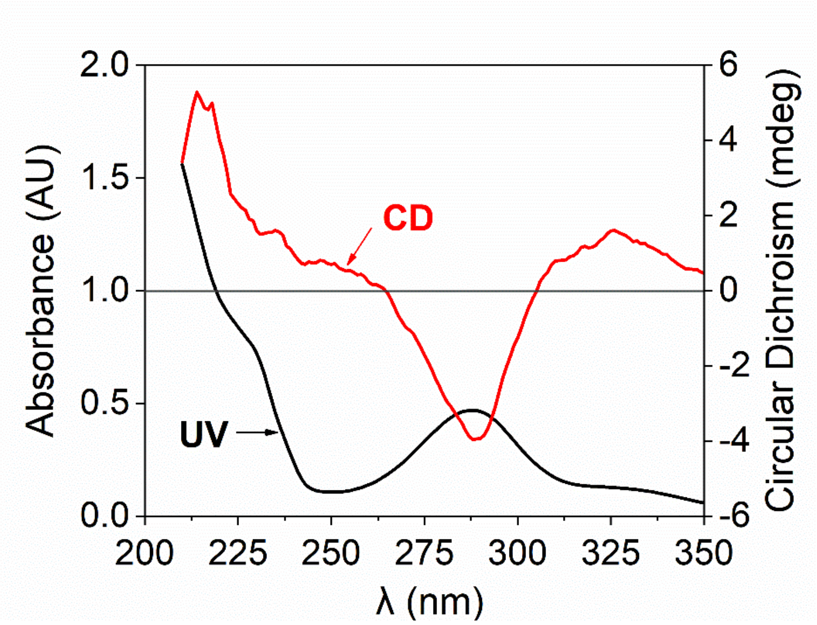

Supplementary Figure 16 The UV (black line) and circular dichroism (CD, red line) spectra of the separated *S*-(–)-naringenin in methanol solution.

Supplementary References

1. Steinkellner, G. et al. Identification of promiscuous ene-reductase activity by mining structural databases using active site constellations. *Nat. Commun.* **5**, 4150 (2014).
