# Supplementary material for: Discovery of an ene-reductase for initiating flavone and flavonol catabolism in gut bacteria
Source: Nat Commun. 2021 Feb 4;12:790. doi: 10.1038/s41467-021-20974-2 (PMC7862272; doi:10.1038/s41467-021-20974-2)
Supplement: Supplementary file 3 — Reporting summary [file 41467_2021_20974_MOESM3_ESM.pdf]

nature research

Corresponding author(s): Yang Gu

Last updated by author(s): Dec 13, 2020

Reporting Summary

Nature Research wishes to improve the reproducibility of the work that we publish. This form provides structure for consistency and transparency in reporting. For further information on Nature Research policies, see Authors & Referees and the Editorial Policy Checklist .

Statistics

For all statistical analyses, confirm that the following items are present in the figure legend, table legend, main text, or Methods section.

n/a	Confirmed	
☐	☒	The exact sample size (*n*) for each experimental group/condition, given as a discrete number and unit of measurement
☐	☒	A statement on whether measurements were taken from distinct samples or whether the same sample was measured repeatedly
☐	☒	The statistical test(s) used AND whether they are one- or two-sided<br>*Only common tests should be described solely by name; describe more complex techniques in the Methods section.*
☒	☐	A description of all covariates tested
☐	☒	A description of any assumptions or corrections, such as tests of normality and adjustment for multiple comparisons
☐	☒	A full description of the statistical parameters including central tendency (e.g. means) or other basic estimates (e.g. regression coefficient) AND variation (e.g. standard deviation) or associated estimates of uncertainty (e.g. confidence intervals)
☐	☒	For null hypothesis testing, the test statistic (e.g. *F*, *t*, *r*) with confidence intervals, effect sizes, degrees of freedom and *P* value noted<br>*Give P values as exact values whenever suitable.*
☒	☐	For Bayesian analysis, information on the choice of priors and Markov chain Monte Carlo settings
☒	☐	For hierarchical and complex designs, identification of the appropriate level for tests and full reporting of outcomes
☒	☐	Estimates of effect sizes (e.g. Cohen's *d*, Pearson's *r*), indicating how they were calculated

Our web collection on statistics for biologists contains articles on many of the points above.

Software and code

Policy information about availability of computer code

Data collection	LC-MS data were collected using the 1290 Infinity HPLC coupled with a 6545 Q-TOF mass spectrometer detector (Agilent, Waldbronn, Germany) and Mass Hunter Workstation version B.08.00 respectively. The data of native FLR crystals were collected at Shanghai Synchrotron Radiation Facility (SSRF) beamline BL17U and were processed using HKL3000. SeMet-labelled FLR, FLR-chrysin, FLR-apigenin and FLR-luteolin crystals were collected at SSRF beamline BL19U.TqRT-PCR experiments were performed by using Bio-Rad iQ5 real-time PCR detection system (Bio-Rad, Palo Alto, USA). Cary 60 UV-Vis spectrophotometer (Agilent Technologies, Santa Clara, USA) was used to record UV-Vis spectrum of the FLR and FMN standard.
Data analysis	Enzyme assay analysis and relative quantification of chemical abundance were conducted by using Origin (version 8.0) software. The enzymatic kinetics data and statistics analysis were conducted by using Graphpad (version 7.0) software. LC-MS data analysis were conducted by using Mass HunterWorkstation (version B.08.00). Sequences of ene-reductase were downloaded from KEGG database using Python (version 3.6.8) script. All structure models were solved and refined with Phenix (version 1.10-2155) and manually built using Coot (version 0.8.2). Sequence similarity networks were analyzed with the publicly available web tools (https://efi.igb.illinois.edu), and visualized by Cytoscape (version 3.5.1) as described in the Methods. The resulting proteins were aligned using the Clustal W (version 2.0) software. MEGA-X (version 10.0.2) software was used to construct the phylogenetic tree. PCR-primer design was carried out by using the SP Designer (version 6.5.0). Specificities of primers for strains were confirmed in silico by using the Primer-BLAST online software. The acquired CD spectra were converted to mean residue ellipticity by using the Pro-Data viewer (version 4.0) software.

For manuscripts utilizing custom algorithms or software that are central to the research but not yet described in published literature, software must be made available to editors/reviewers. We strongly encourage code deposition in a community repository (e.g. GitHub). See the Nature Research guidelines for submitting code & software for further information.

Data

Policy information about availability of data

All manuscripts must include a data availability statement. This statement should provide the following information, where applicable:
- Accession codes, unique identifiers, or web links for publicly available datasets
- A list of figures that have associated raw data
- A description of any restrictions on data availability

The atomic coordinates of FLR-apo, FLR-chrysin, FLR-apigenin, and FLR-luteolin structures have been deposited in the Protein Data Bank with accession codes of 7D39, 7D38, 7D3A, and 7D3B, respectively. Other relevant data supporting the findings of this research are available in the article and the Supplementary Information. In addition, datasets generated and analyzed in the study are available from the corresponding author upon reasonable requests. The amino acid sequences of enzymes (containing EC numbers) for SSN analysis were downloaded from KEGG. The generated source code is available at https://github.com/lovingstudy/kegg_api/blob/master/extract_aaseq_kegg.py (in Python programming language, v3.6.8). The Metaquery database (http://metaquery.docpollard.org/) was used to assess the metagenomic abundance of flr homologues. Raw data are supplied in Source Data file. In addition, datasets generated and analyzed in the study are available from the corresponding author upon reasonable requests.

Field-specific reporting

Please select the one below that is the best fit for your research. If you are not sure, read the appropriate sections before making your selection.

☒ Life sciences ☐ Behavioural & social sciences ☐ Ecological, evolutionary & environmental sciences

For a reference copy of the document with all sections, see nature.com/documents/nr-reporting-summary-flat.pdf

Life sciences study design

All studies must disclose on these points even when the disclosure is negative.

Sample size	Samples size for each experiment is indicated in the legends. No statistical methods were used to predetermine sample size. The samples size (triplicates) for microbial fermentations was chosen based on the common approach in this field. For testing enzymatic activity, we used triplicates because this is generally accepted. For all chromatograms and mass spectra, data shown are representatives of at least three biological replicates according to accepted methods in the field.
Data exclusions	No data were excluded.
Replication	All experiments were successfully repeated at least three times, and the number of independent experiments or biological replicates is indicated in the figure legends. Purification of proteins were performed without repeating. For all data showing representative chromatograms and mass spectra, at least three biological replicates were performed.
Randomization	Samples were randomly assigned to the treatment and control groups with no formal randomization techniques.
Blinding	This study includes bioinformatics analysis, enzymatic reactions, microbial fermentations, and structural dissections; blinding is not relevant to these experiments.

Reporting for specific materials, systems and methods

We require information from authors about some types of materials, experimental systems and methods used in many studies. Here, indicate whether each material, system or method listed is relevant to your study. If you are not sure if a list item applies to your research, read the appropriate section before selecting a response.

Materials & experimental systems

n/a	Involved in the study	
☒	☐	Antibodies
☒	☐	Eukaryotic cell lines
☒	☐	Palaeontology
☒	☐	Animals and other organisms
☒	☐	Human research participants
☒	☐	Clinical data

Methods

n/a	Involved in the study	
☒	☐	ChIP-seq
☒	☐	Flow cytometry
☒	☐	MRI-based neuroimaging
